# Generation of Megakaryocytic Progenitors from Human Embryonic Stem Cells in a Feeder- and Serum-Free Medium

**DOI:** 10.1371/journal.pone.0055530

**Published:** 2013-02-12

**Authors:** Marjorie Pick, Lisa Azzola, Elissa Osborne, Edouard G. Stanley, Andrew G. Elefanty

**Affiliations:** 1 Monash Immunology and Stem Cell Laboratories, Monash University, Clayton, Victoria, Australia; 2 Monash Reproductive Pathology and Genetics, Monash IVF, Clayton, Victoria, Australia; Johns Hopkins School of Medicine, United States of America

## Abstract

**Background:**

The production of human platelets from embryonic stem cells in a defined culture system is a prerequisite for the generation of platelets for therapeutic use. As an important step towards this goal, we report the differentiation of human embryonic stem cells (hESCs) towards the megakaryocyte (Mk) lineage using a ‘spin embryoid body’ method in serum-free differentiation medium.

**Methodology and Principal Findings:**

Immunophenotypic analyses of differentiating hESC identified a subpopulation of cells expressing high levels of CD41a that expressed other markers associated with the Mk lineage, including CD110, CD42b and CD61. Differentiated cells were sorted on the basis of their expression of CD41a, CD34 and CD45 and assessed for Mk colony formation, expression of myeloid and Mk genes and ability to endoreplicate DNA. In a collagen-based colony assay, the CD41a^+^ cells sorted from these differentiation cultures produced 100–800 Mk progenitors at day 13 and 25–160 Mk progenitors at day 20 of differentiation per 100,000 cells assayed. Differentiated Mk cells produced platelet-like particles which expressed CD42b and were activated by ADP, similar to platelets generated from precursors in cord blood. These studies were complemented by real time PCR analyses showing that subsets of cells enriched for CD41a^+^ Mk precursors expressed high levels of Mk associated genes such as *PF4* and *MPL*. Conversely, high levels of myeloid and erythroid related transcripts, such as *GATA1*, *TAL1/SCL* and *PU.1*, were detected in sorted fractions containing CD34^+^ and CD45^+^ cells.

**Conclusions:**

We describe a serum- and feeder-free culture system that enabled the generation of Mk progenitors from human embryonic stem cells. These cells formed colonies that included differentiated Mks that fragmented to form platelet-like particles. This protocol represents an important step towards the generation of human platelets for therapeutic use.

## Introduction

Pancytopenia and thrombocytopenia remain significant clinical problems for patients with a range of medical conditions, especially those undergoing repeated cycles of chemotherapy as treatment for cancer. Since finding suitable HLA-matched volunteers to donate platelets for these patients can be difficult, it is prudent to explore alternative sources for such blood products. The ability to differentiate hematopoietic lineages from human embryonic stem cells (hESCs) or induced pluripotent stem cells represents a potential alternate supply of megakaryocytes (Mks) and platelets for research and eventually for the treatment of thrombocytopenia. Whilst Mks have been generated from mouse ESCs [1]–[3] and hESCs [4]–[8], many of the prior differentiation protocols have included co-culture with stromal cells which makes it difficult to delineate the exact requirements needed for direct differentiation of Mks. We have established a two step serum-free and stromal cell-free differentiation protocol, initially generating hematopoietic progenitor cells that express CD34, CD41a (hereafter abbreviated to CD41) and CD45 and subsequently differentiating these progenitors towards CD41-positive Mks.

Our laboratory has established protocols whereby homogenous ‘spin embryoid bodies (EBs)’ generated from hESCs [9] were supplemented with bone morphogenetic protein (BMP4), stem cell factor (SCF), vascular endothelial growth factor (VEGF) and basic fibroblast growth factor (FGF2) for 10 days (d) to produce hematopoietic progenitors [10]. In our current studies we differentiated hESCs in serum-free medium supplemented with growth factor combinations designed to support the generation of Mks from hematopoietic mesoderm. We showed that the Mk progenitors were found in CD41^+^ and CD34^+^ cell fractions when assayed at d13 and d20 of differentiation. In collagen-based assays, differentiated progeny of CD41^+^ Mk colony forming cells appeared to shed pro-platelets. Real time PCR analyses showed that these subsets of cells expressed high levels of Mk associated genes such as *PF4* and *MPL*, whilst other cell fractions were enriched for cells expressing myeloid or endothelial-associated genes. Finally, ploidy analyses suggested that d20 CD41^+^CD34^lo^CD45^+^ cells contained a higher proportion of polyploid cells than CD41^lo/−^CD34^lo^CD45^+^ counterparts.

## Materials and Methods

### hESC Culture and Differentiation

Human embryonic stem cells (HES3 [11], Envy [12] and MEL1 (Millipore, Billerica, MA, USA)) were maintained on irradiated mouse embryonic fibroblast (MEF) feeder cells in DMEM/Hams F12 medium supplemented with 20% knockout serum replacer (Invitrogen) and 4 ng/ml recombinant human (rh)FGF2 (Peprotech, Rocky Hill, NJ) by mechanical passaging [10]. In order to generate cells for differentiation experiments, colonies were expanded onto tissue culture flasks pre-seeded with irradiated MEFs and adapted to enzymatic passaging with TrypLE Select (Invitrogen) as previously described [10], [13].

On the day prior to differentiation, hESCs were passaged onto tissue culture flasks seeded with irradiated (30 Gy) MEFs and the cultures were harvested with TrypLE Select (Invitrogen). Single cells were resuspended in serum-free BPEL medium [14] supplemented with 5–15 ng/ml rhBMP4 (R&D systems, Inc., Mn), 10–15 ng/ml rhVEGF (Peprotech), 10 ng/ml rhFGF2 (Peprotech) and 25 ng/ml rhSCF (Peprotech) as described previously [10]. One hundred microlitres of cell suspension containing 2,500 hESCs was deposited into each well of round bottomed low adherence 96 well plates.

Within 24 hours, single embryoid bodies (EBs) formed in each well. After 10 days, 72 EBs were transferred to each well of a 6 well flat-bottom tissue culture plate containing 7.2 ml of BPEL supplemented with 20 ng/ml rh thrombopoietin (TPO), 25 ng/ml rhSCF and 25 ng/ml rh interleukin (IL)-3 (Peprotech). Cells were cultured for a total of 13 or 20 days after which time the EBs were disaggregated, stained with the appropriate antibodies and analyzed or sorted by flow cytometry. The hESC lines used in this experiment were monitored for retention of a normal karyotype.

### Flow Cytometric Analysis and Sorting of Differentiated hESCs

EBs generated from differentiated hESCs were harvested, disaggregated and stained at d13 with anti-CD34-PE (BD Biosciences) and anti-CD41-APC (BD Biosciences) monoclonal antibodies. At d20 of differentiation hESCs were stained with CD34-FITC (BD Biosciences), CD45-PE (BD Biosciences) and CD41-APC while the GFP expressing Envy cells were stained with CD45-PE, CD34-PerCP (BD Biosciences) and CD41-APC. All antibody concentrations were titrated to give optimal specific staining and incubated with cells for 30 minutes at 4°C. Cells were washed in phosphate buffered saline (PBS), centrifuged at 480 g for 5 minutes at 4°C and cell pellets were resuspended in PBS containing propidium iodide (PI) to exclude dead cells and analyzed on a FACSCalibur using CellQuest Pro software (BD Biosciences). For cell sorting experiments, 4′, 6-diamidino-2-phenylindole (DAPI) exclusion was used to identify viable cells. Multi-color cell sorting (FITC, PE, PerCP, APC, DAPI) using three separate lasers (488 nM, 630 nM and UV) was used to gate and sort cells into four or five populations using a FACSVantage flow cytometer (BD Bioscience**)**.

### Hematopoietic Colony Forming Assays

#### Methylcellulose-based myeloid colony forming assay

Triplicate assays were performed in 24 well tissue culture treated plates (Nunc) with 10,000 cells added per well in 0.5 mL of Methocult™ (Stem Cell Technologies, Canada) supplemented with 20 ng/ml rh granulocyte-macrophage-colony stimulating factor (GM-CSF), 50 ng/ml rhSCF, 20 ng/ml rhIL-3, 3 U/ml rh erythropoietin (EPO) and 20 ng/ml rhIL-6 (all from Peprotech). Cultures were incubated at 37°C in 5% C0_2_ and scored for colony formation after 14 days.

#### Collagen-based megakaryocyte colony forming assay

Duplicate assays were performed in 24 well tissue culture treated plates into which 10,000 cells were mixed with a collagen based medium (MegaCult™, Stem Cell Technologies) supplemented with 50 ng/ml rhTPO, 25 ng/ml rhSCF and 10 ng/ml rhIL-3 to stimulate colony formation. After 14 days plates were dried and stained with anti-CD41 antibody. Positive cells were detected using an alkaline phosphatase colorimetric assay (Stem Cell Technologies).

### Quantification of Nucleii in Megakaryocytes

Cytospin preparations of day 13 and day 20 cultures of differentiated cells were analyzed for the number of nucleii present in the megakaryocytes generated. Staining with May Grunwald-Giemsa allowed easy identification of the megakaryocytes and quantitation of nucleii per megakaryocyte within each sorted fraction.

### Quantification of Platelet Production from CFU-Mk Colonies

After staining the CFU-Mk colonies using the MegaCult™ kit, the percentage of CD41^+^ colonies producing CD41^+^ platelet-like particles was calculated. Each colony was also scored for the number of CD41^+^ cells within the colony that generated CD41^+^ platelet-like particles.

### Flow Cytometric Analysis of Platelet-like Particles

To harvest platelet-like particles from cultures, the supernatants from day 20 differentiated hESCs were centrifuged at 480 g from 5 min at 4°C and the pelleted particles were resuspended in PBS. These were incubated with the directly conjugated antibodies CD41a-FITC and CD42b-PE. Flow cytometry data was gated using log amplification of forward and side scatter to include the small platelet-like particles, using platelets generated from umbilical cord blood as a positive control [15]. Isotype controls for the antibody stains were included. Data was acquired on a FACSCalibur (BD Biosciences). Cord blood samples, received from a private source with appropriate ethics approval, were used as normal controls.

### Platelet Activation

Platelet-like particles were harvested from day 20 differentiated cultures as described above, pelleted by centrifugation and resuspended in PBS. The pelleted particles were incubated in the presence or absence of 20 nM ADP at room temperature form 20 min and stained for the expression of CD41a-FITC and CD62P-PE [15]. Platelets generated from umbilical cord blood were included as a positive control [15]. Isotype controls for the antibody stains were included. Data was acquired on a FACSCalibur (BD Biosciences). Cord blood samples, received from a private source with appropriate ethics approval, were used as normal controls.

### Quantitative Real Time PCR

Total RNA from undifferentiated and differentiated hESCs was prepared using RNEasy reagents according to the manufacturer’s instructions (Qiagen). First-strand cDNA was reverse transcribed with random hexamer priming using Superscript III reagents (Invitrogen). Real-time PCR was performed using Taqman gene expression probes (Applied Bioscience), the 7500 Fast Real-time PCR system absolute thermal cycler and software (Applied Bioscience). The comparative cycle threshold (CT) method was used to analyze data, with gene expression levels compared to GAPDH expression as previously described [10]. In brief, the CT for expression was calculated for each gene and for GAPDH. Since gene expression is inversely proportional to the CT, the expression for a given target gene relative to GAPDH may be given by the formula: 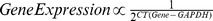. In this paper, this calculated value was multiplied by 1000 for the purposes of presentation.

### Analysis of Cell Ploidy

Cell ploidy in sorted fractions was analyzed by staining cellular DNA with PI. Prior to analysis, flow sorted cells were fixed in cold ethanol at 4°C overnight. Cells were pelleted and resuspended in PBS containing RNase A (Sigma) and incubated for 30 minutes at 37°C. Prior to analysis, PI (Sigma) was added. Samples were analyzed on a FACSCalibur using CellQuest Pro software (Becton Dickinson).

### Fluorescence *in situ* Hybridization (FISH)

To detect cells with ≥4 N DNA, sorted cell fractions were analysed using fluorescence in situ hybridization (FISH). Sorted cells were resuspended in fixative (3∶1 methanol:glacial acetic acid) and an aliquot of the cell suspension was dropped onto a glass slide and left to dry. Samples were dehydrated through a series of ethanol solutions (75%, 90%, 100%), dried and stored at −20°C. Three FISH probes were used for analysis, namely CEP15 (aqua) detecting chromosome 15, CEP16 (orange) detecting chromosome 16 and LSI22 (q11.2) (green) detecting chromosome 22 (Vysis, Immunodiagnostics, Victoria, Australia). Probe mixture (1.5 µl) was applied to each slide and coverslipped. Slides were denatured at 73°C for 5 minutes and incubated at 37°C for a further 3 hours. The coverslip was removed and the slides were washed in 0.4× Sodium Chloride Sodium Citrate (SSC) at 71°C for 30 seconds then for a further 2 minutes at room temperature. Slides were air-dried and counterstained with DAPI (Vysis). Slides were analyzed under 400× and 1000× magnification using an Olympus BX51 fluorescent microscope (Olympus) and imaged using Quips Imaging Software, version 3.1.2 (Vysis).

## Results

### Expression Profile of CD41 on Hematopoietic Cells Generated from Differentiated hESCs

As a first step in the identification of a cell population enriched for Mk progenitors, we surveyed the expression of CD41 (GpIIb), a surface molecule expressed on most early hematopoietic progenitor cells [16]–[19], on differentiating hESCs. hESCs were cultured for 10 d in serum free medium supplemented with BMP4, VEGF, SCF and FGF2 to induce mesoderm and commit cells to hematopoiesis and then for a further 3 d or 10 d in medium containing TPO, SCF and IL-3 in order to promote megakaryopoiesis.

**Figure 1:**
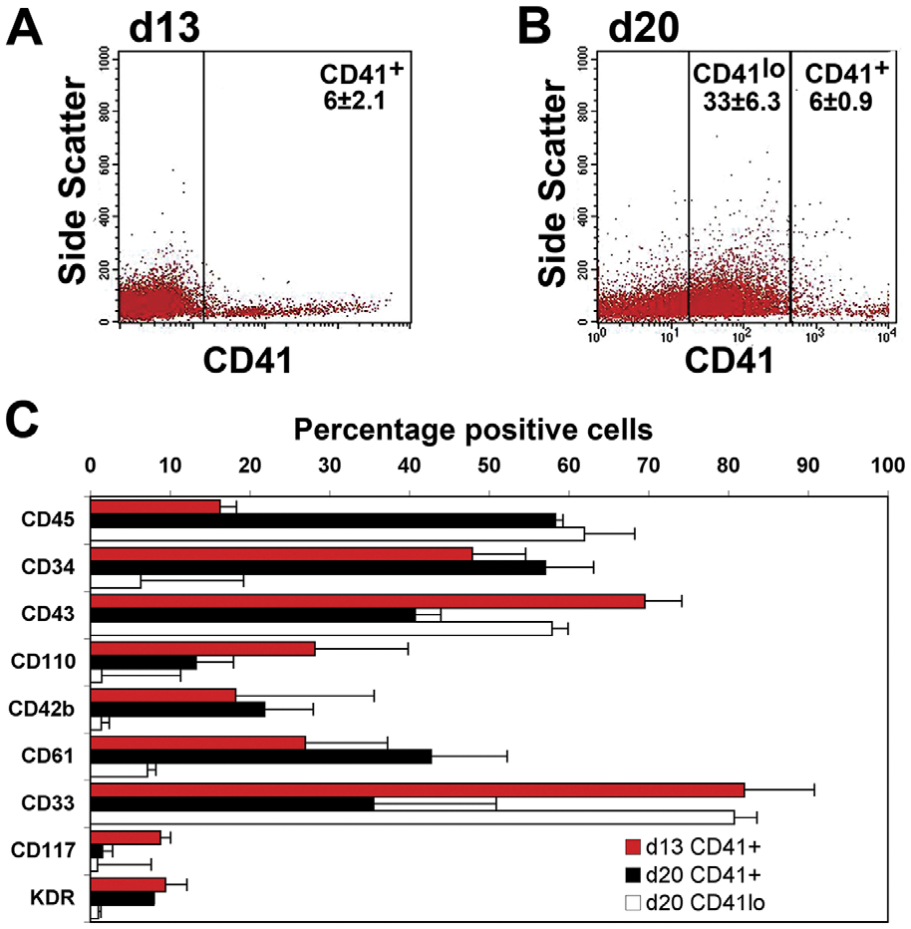
Immunophenotype of CD41 positive cells in human embryonic stem cell differentiation cultures. Differentiating hESCs were cultured for 10 days (d) in BMP4, VEGF, SCF and FGF2 and then for a further 3 or 10 d in TPO, SCF and IL-3. Cells were dissociated at d13 or d20 of culture and stained with antibodies directed against CD41 and a panel of hematopoietic markers. A) Gating strategy identifying CD41^+^ cells at d13 and B) CD41^lo^ and CD41^+^ cells at d20 of hESC differentiation. C) Percent expression of hematopoietic - CD45, CD34, CD43; megakaryocytic – CD110, CD42b, CD61, myeloid – CD33 and hemato/endothelial – CD117 and KDR surface markers on the CD41 expressing cells. (Representative FACS panels stained with isotype controls for CD41 staining are shown in Figure S1).

After 13 and 20 days of differentiation, the expression of CD41 was examined in combination with the expression of a panel of cell surface markers associated with hematopoietic and endothelial cells (Figure 1 and Figure S1). At d13, a medium to bright CD41^+^ population was observed (6±2.1%, Figure 1A), while at d20 the CD41 expression could be subdivided into CD41^+^ (6±0.9%) and a CD41^lo^ (33±6.3%) populations (Figure 1B). The majority (∼70%) of CD41^+^ cells at d13 expressed markers of immature hematopoietic cells and their progenitors (CD34, CD43 and CD33), ∼20% expressed hematopoietic and Mk markers (CD45, CD110 (MPL), CD42b and CD61), and less than 10% expressed CD117 (KIT) or KDR, molecules observed on both hematopoietic progenitor cells and endothelium (Figure 1C). Analysis of CD41 expression at d20 revealed that over 50% of the CD41^+^ cells retained expression of CD34 and a higher proportion now expressed CD45 and CD61, consistent with ongoing Mk maturation. In contrast, very few of the CD41^lo^ cells continued to express CD34 but an increase in CD43, CD45 and CD33 expressing cells was observed suggesting differentiation to non-megakaryocytic myeloid lineages (Figure 1C).

**Figure 2:**
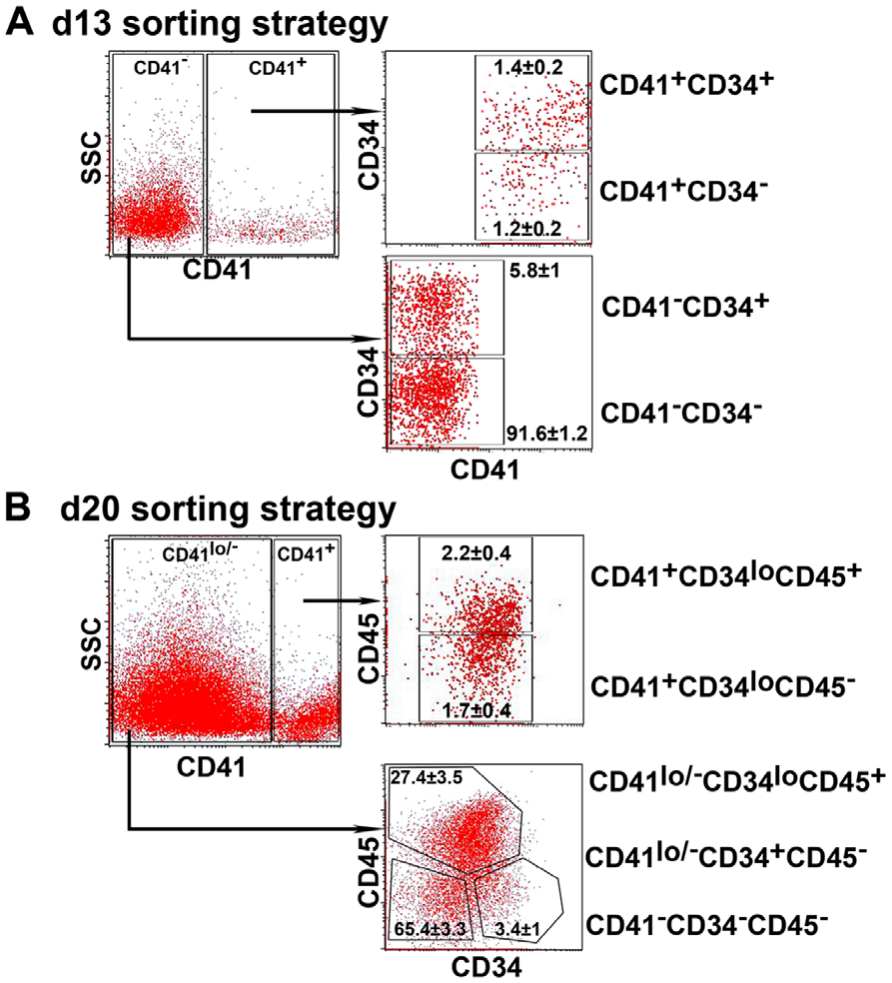
Flow sorting strategy for the day 13 and day 20 differentiated human embryonic stem cells. Cells were sorted according to their expression of CD41 and CD34 on day (d)13 and CD41, CD34 and CD45 on d20. (A) Gating strategy used to sort 4 fractions on d 13 and (B) 5 fractions on d20. Cells that were CD34^−^ CD45^−^ at d 20 were also CD41^−^. The percent distribution (mean±SD) for each sorted fraction is shown (n = 5).

Embryoid bodies from differentiating hESC lines were harvested at d13 and d20 and sorted by flow cytometry based on their expression of CD41, CD45 and CD34 (Figure 2). Most experiments were performed with HES3 cells but similar results were obtained with Envy and MEL1 lines (Table S1 and data not shown). At d13, four fractions were analyzed: CD41^+^CD34^+^, CD41^+^CD34^−^, CD41^−^CD34^+^, and CD41^−^CD34^−^ (Figure 2A); while d20 differentiated cells were sorted into five fractions: CD41^+^ CD34^lo^ CD45^+^, CD41^+^ CD34^lo^ CD45^−^, CD41^lo/−^ CD34^lo^ CD45^+^, CD41^lo/−^CD34^+^CD45^−^ and CD41^−^CD34^−^CD45^−^ (Figure 2B).

### Megakaryocytic Colonies Generated in Collagen-based Cultures

**Figure 3:**
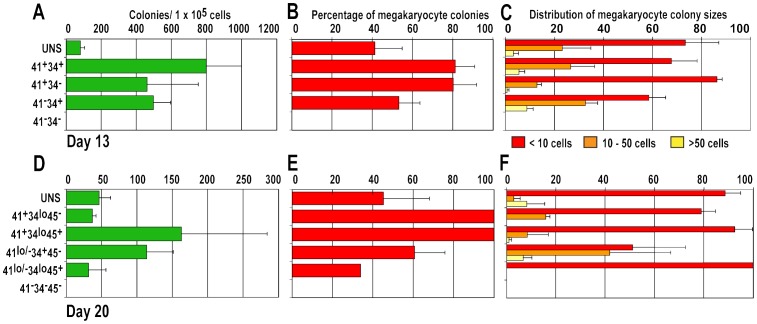
Analysis of megakaryocytic colonies generated from human embryonic stem cell differentiation cultures. Cells sorted according to their expression of CD41 and CD34 on day (d)13 and CD41, CD34 and CD45 on d20 were plated in Megacult semi solid medium and colonies scored after 14 days. The total number of colonies (A, D) and the proportion of CD41^+^ megakaryocytic colonies (B, E) in d13 (A, B) and d20 (D, E) samples are shown. The distribution of megakaryocytic colony size is shown (C, F). Results shown represent the mean±SEM of 4 experiments. At d13, the frequency of small (<10 cells) and medium (10–50 cells) megakaryocytic colonies differed significantly between CD41^+^CD34^−^ and CD41^−^CD34^+^ fractions (p<0.05 and p<0.02 for small and medium colonies, respectively).

To determine the capacity of the sorted fractions to generate Mk colonies, cells were plated in a collagen-based semi-solid culture medium. After 14 days, the cultures were fixed and Mk detected by staining with anti-human CD41a. Colony-forming cells (CFCs) at d13 of differentiation were confined to the CD41^+^ and CD34^+^ fractions (Figure 3A) with the majority of colonies containing CD41^+^ Mk cells (Figure 3B). The Mk colonies generated from the CD41^−^CD34^+^ sorted fraction were larger than those generated from the CD41^+^CD34^−^ sorted fractions (Figure 3C).

MK-CFCs at d20 of differentiation were enriched in two sorted fractions, CD41^+^CD34^lo^CD45^+^ and the CD41^lo/−^ CD34^+^CD45^−^ (Figure 3D), with the highest proportion of Mk colonies generated in the sorted fractions that contained CD41^+^ cells (Figure 3E). Similar to observations in d13 colonies, the fraction containing CD34^+^ cells tended to generate the largest Mk colonies, although the differences between fractions did not reach statistical significance in these experiments (Figure 3F).

**Figure 4:**
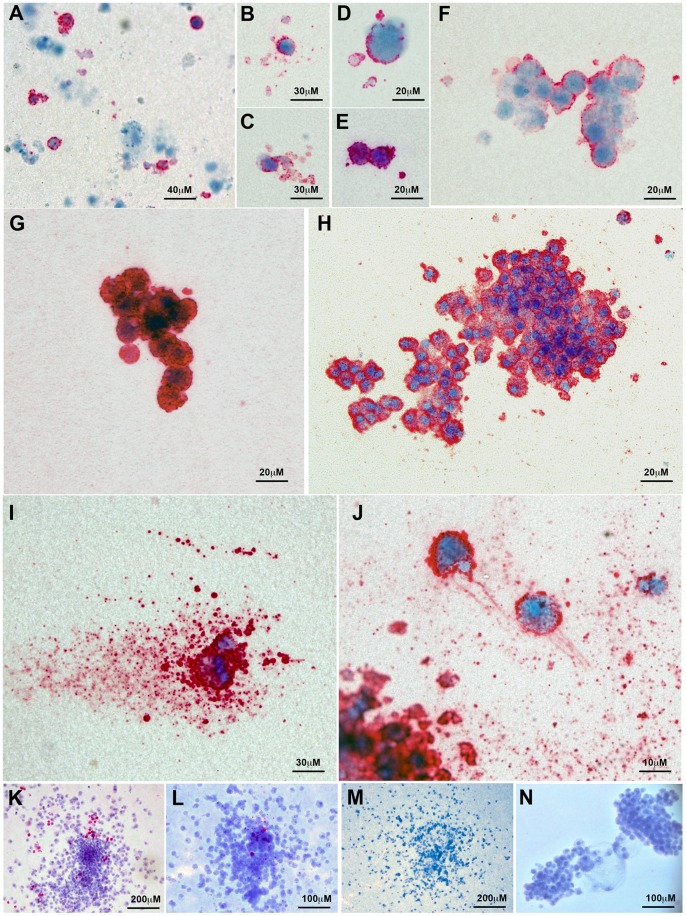
Images of megakaryocytic colonies expressing CD41 cultured from differentiated human ES cells. Images of colonies were taken from both day 13 and 20 assays. (A) Image showing both CD41-positive and -negative cells within the culture. (B–E) CD41^+^ cells surrounded by CD41^+^ cellular fragments, possibly representing platelets. Small (F) weakly and (G) strongly staining CD41^+^ colonies. (H) Large CD41^+^ colony (I) Single polyploid CD41^+^ cell generating many platelet like fragments (J) Section of a Mk colony showing two CD41^+^ cells and many platelet like fragments, (K–L) Mixed colonies comprising predominantly non megakaryocytic cells (M, N) Two CD41-negative colonies.

Single CD41-positive cells were seen dispersed throughout the cultures and, in a number of cases, these were surrounded by platelet-like CD41^+^ cellular fragments of variable size (Figure 4B–E). Indeed, some polyploid cells appeared to be producing thousands of platelet-like fragments (Figure 4I). Mk colonies varied in size and CD41 staining intensity (Figure 4F–H). Depending on the population of sorted cells, myeloid colonies were evident that contained few or no CD41^+^ cells (Figure 3B, 3E and Figure 4K–L).

Sorted cells from each fraction were also plated in methylcellulose cultures supplemented with cytokines to stimulate myeloid and erythroid colony growth. It was noteworthy that the highest frequencies of erythroid CFCs in cells sorted from differentiation cultures at d13 were in the CD41^+^ fractions that also generated the highest proportion of Mk colonies. Similarly, the CD41^+^CD34^lo^CD45^+^ fraction that yielded the highest number of Mk colonies from d20 differentiated cultures, also generated erythroid colonies with the greatest frequency (Figure S2). These data are consistent with the finding that Mk and erythroid colonies generated from hESCs may share a common precursor [7], [20]. Colony forming activity was always confined to cells expressing either CD41 and/or CD34, with the frequency of CFCs higher at d13 (142±162 CFU/10^5^ cells plated) than at d20 (65±56 CFU/10^5^ cells plated) (Figure S2).

### Platelet-like Particles Produced by Megakaryocytes Express CD42b and Respond to ADP

**Figure 5:**
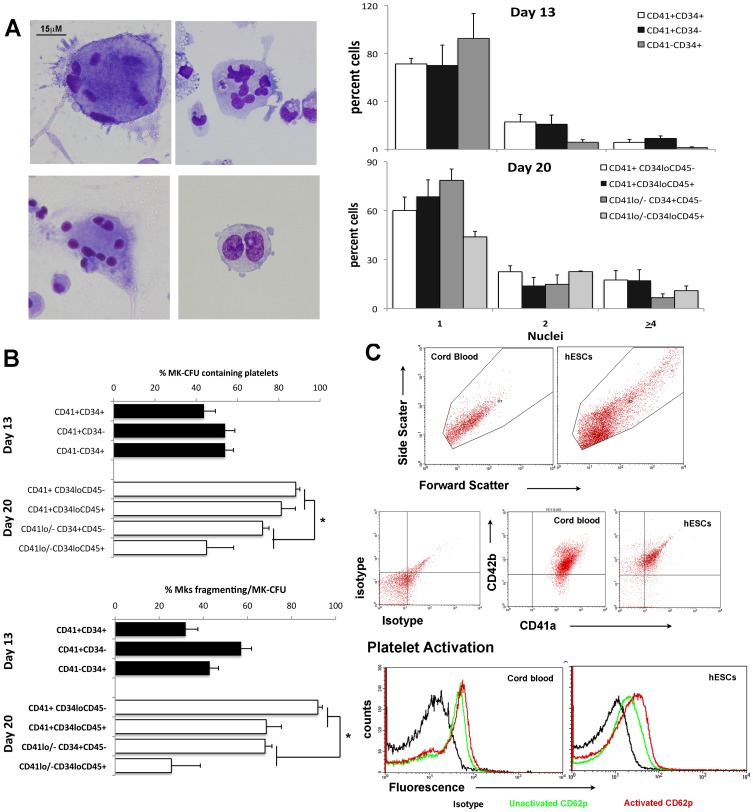
Platelet-like particles generated from human embryonic stem cells (hESCs). A) May-Grunwald-Giemsa staining of megakaryocytes (Mk) showing endoreplication and membrane blebbing after 13 and 20 days of differentiation of hESCs. The frequency of cells containing 1, 2 or ≥4 nucleii was quantified from stained preparations of single cells at day 13 and 20 of differentiation and results are shown as mean±SEM of n = 4–8 experiments. B) Mk colonies (CFU-Mk) were generated after 13 and 20 days of differentiation by placing differentiated cells in Megacult. The numbers of platelet-like particles were quantitated per CD41^+^ Mk colony. The upper histogram shows the percentage of CFU-Mks that produced platelet-like particles whilst the lower histogram depicts the percentage of Mks generating platelet-like particles per CD41^+^ colony. Results shown represent the mean±SEM of n = 4–8 experiments. *, p≤0.05 between the CD41^+^ sorted fractions and CD41^−/lo^ sorted fractions generated in the day 20 differentiated cells. C) Flow cytometric analysis of platelet-like particles generated from cord blood and hESCs gated on forward scatter and side scatter, showing that the platelet-like particles expressed both CD41a and CD42b, similar to cord blood derived platelets. The lower histogram shows increased expression of the activation marker CD62p in response to incubation with 20 uM of ADP (data from one of two similar experiments is shown).

Polyploid nucleii arising as a result of endomitosis are a unique characteristic of maturing Mks and a higher number of nucleii is predictive for greater platelet production. Polyploid megakaryocytes with membrane blebbing or fragmentation, suggests platelet formation, were observed in cytospins of CD41^+^ sorted fractions from day 13 and 20 differentiation cultures (Figure 5A). Consistent with this, approximately 50% of Mk colonies derived from day 13 progenitors produced platelet-like particles (Figure 5B). A greater percentage of the Mk colonies generated from CD41^+^ sorted fractions at day 20 were associated with platelet-like particles than was seen in Mk colonies generated from the CD41^lo/−^ sorted fractions (84.7±15.2% versus 58.6±41.2%)(Figure 5B). Furthermore, the percentage of Mks in each colony generating platelet-like particles was significantly higher in colonies generated from the CD41^+^ than from the CD41^lo/−^ sorted fractions (80.3±4.4% versus 46.8±8.1%) (Figure 5B).

The platelet-like particles were harvested from the supernatants of the differentiating hESCs at day 20 and analyzed by flow cytometry. Their forward and side scatter profiles were generally similar to platelets generated form cord blood, although the hESC-derived platelet-like particles appeared to encompass a greater range of particle sizes (Figure 5C). However, particles from hESCs and cord blood derived platelets expressed both CD41a and CD42b and upregulated the expression of CD62P, a marker for platelet activation, in response to incubations with ADP (Figure 5C).

### Increased Expression of Megakaryocytic Marker Genes in CD41^+^ Sorted Fractions

**Figure 6:**
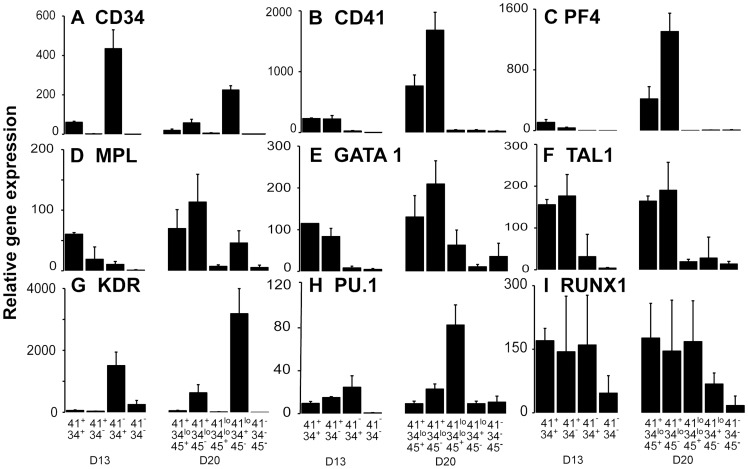
Hematopoietic gene expression in differentiated hESCs. cDNA was generated from fractions sorted on the basis of CD34, CD41 and CD45 expression at day 13 and 20 of differentiation. Gene expression was quantified by real time-PCR analysis for the indicated genes. Histograms show relative gene expression expressed as mean±sSEM for 3–9 independent experiments.

Real time PCR analysis was used to quantify gene expression in fractions sorted from d13 and d20 cultures (Figure 6). Both *CD34* and *CD41* were more highly transcribed in fractions sorted on those surface proteins, although there were large variations in expression level between fractions that generally mirrored the differences in protein expression observed by flow cytometry. Expression of Mk markers such as *PF4* [21] and the thrombopoietin receptor, *MPL*, was significantly enhanced in fractions containing CD41^+^ cells, as was expression of *GATA1* and *TAL1 (SCL),* transcription factors that are co-expressed in Mk and erythroid lineages [22]. *KDR*, encoding the receptor for VEGF, was highly expressed on CD34^+^CD41^−^ and CD41^lo/−^CD34^+^ CD45^−^ fractions. We have shown that cells co-expressing high levels of KDR and CD34 are enriched for endothelial progenitor cells [23]. Expression of the myeloid lymphoid regulator *PU.1* was highest in CD41^−^CD34^+^ and CD41^lo/−^CD34^+^ CD45^+^ sorted cells. In these fractions, levels of *GATA1* were correspondingly lower, consistent with the known mutual inhibition and cross regulation of the two transcription factors [24], [25]. Higher expression of *PU.1* correlated with a reduction in erythroid CFCs (Figure S2) and a reduced proportion of Mk CFCs (Figure 3). High levels of *RUNX1* expression were observed in all fractions with hematopoietic colony forming capacity.

### CD41^+^ Sorted Fractions Contain Cells with High Ploidy

**Figure 7:**
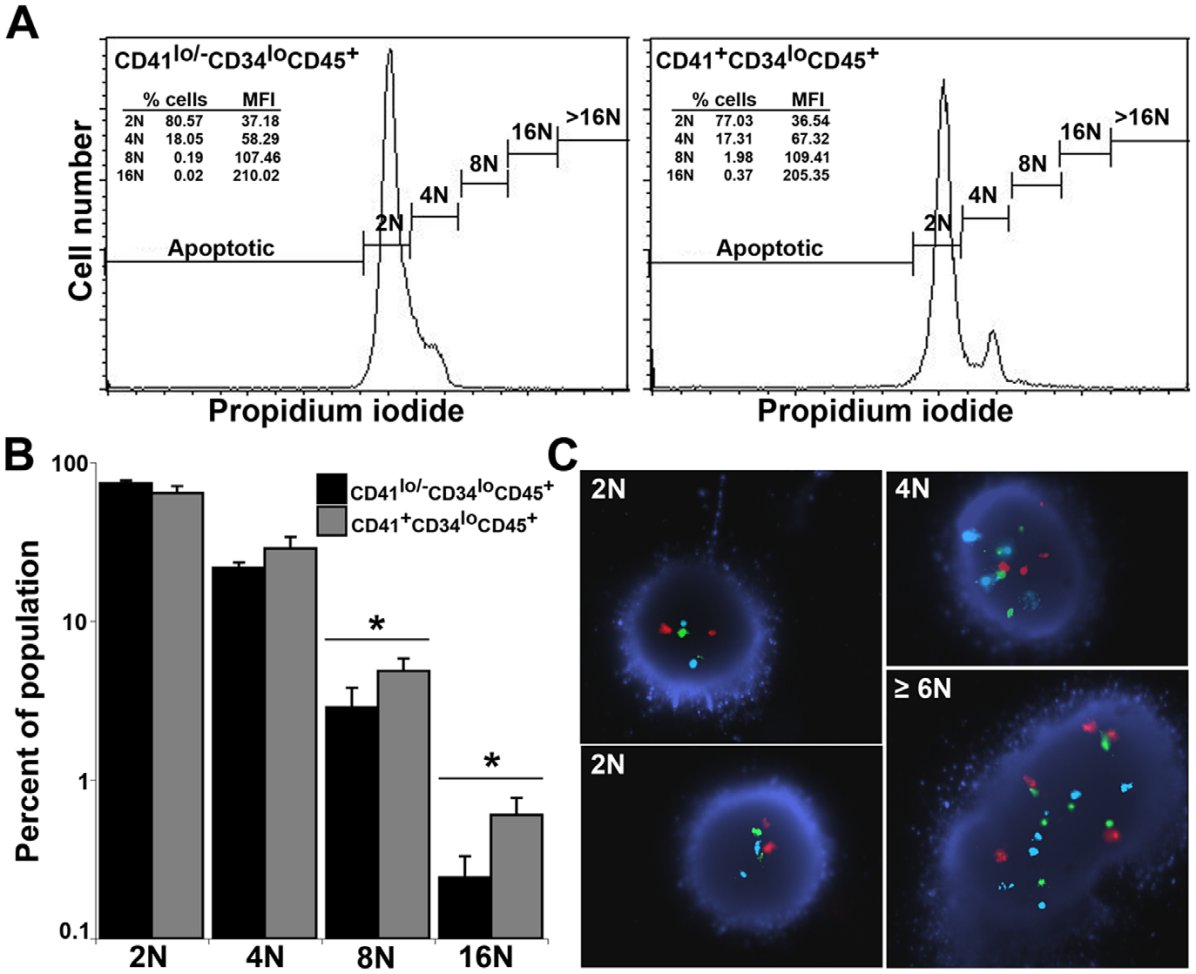
Increased ploidy of day 20 CD41^+^ sorted cells demonstrated by flow cytometry and FISH. (A) Forward scatter flow cytometry profiles of propidium iodide stained CD41^lo/−^CD34^lo^CD45^+^ and CD41^+^CD34^lo^CD45^+^ cells. The proportion of cells corresponding to each ploidy fraction is shown accompanied by the mean fluorescence intensity (MFI). (B) Histogram showing summary of experiments comparing the ploidy of fractions containing CD41^+^ with CD41^lo/−^ cells (mean±sem; n = 4; *, p<0.05) (C) FISH analysis of single cells hybridized with probes against Chromosome (Ch) 15 (aqua), Ch16 (red) and Ch 22 (green) showing examples of 2N, 4N and ≥6N cells.

Megakaryocytes display the ability to replicate DNA without corresponding cell division, in a process called endoreplication, whereby mature cells can reach up to 128N DNA content. Since we were unable to extract live cells in sufficient numbers from the collagen based Mk colony assays, we compared the ploidy between two fractions of sorted cells at d20, CD41^+^CD34^lo^ CD45^+^ and CD41^lo/−^CD34^lo^ CD45^+^, that differed primarily in their expression of CD41. The CD41^+^ cells had displayed a tendency to generate greater numbers of colonies in the collagen based assay than the CD41^lo/−^ cells (163±121 compared with 32±25 Mk colonies per 10^5^ cells, Figure 3), although variability between experiments precluded the results achieving statistical significance. Similarly, a greater proportion of the colonies from CD41^+^ fraction contained Mk cells (100% compared with 34%) (Figure 3). Flow cytometric analysis of DNA content comparing CD41^+^CD34^lo^ CD45^+^ cells with CD41^lo/−^CD34^lo^ CD45^+^ cells, demonstrated that a greater proportion of the CD41^+^fraction contained 8N (4.8±1.9% compared with 2.9±1.9%, p<0.01) or 16N (0.6±0.3% compared with 0.2±0.2%, p<0.05) DNA (Figure 7). Corroborating this finding, FISH analysis using three chromosome probes suggested that a small proportion of cells analyzed from the CD41^+^fraction contained ≥6N DNA, whilst cells from the CD41^lo/−^ fraction only contained 2N and 4N DNA (Figure 7). These experiments suggested that, prior to expansion and maturation of clonogenic precursors in vitro, the CD41^+^CD34^lo^ CD45^+^ fraction contained a small proportion of cells already undergoing endoreplication/endomitosis, consistent with maturation of some Mk precursors in culture.

## Discussion

In these studies we have differentiated human embryonic stem cells towards hematopoietic lineages using our ‘spin EB’ protocol in a serum-free medium [10], [14]. Compared with previously published protocols [26]–[28], we find that the forced aggregation of hESCs generated EBs more uniform in size that differentiated with greater efficiency and synchrony, resulting in the formation of blood cells in over 90% of EBs [9]. The culture medium was supplemented with growth factors that induced and patterned mesoderm to a hematopoietic fate for the first 10 days, followed by a cytokine cocktail designed to bias further differentiation towards the Mk lineage. After 13 and 20 days of differentiation, characterisation of cell populations isolated on the basis of their expression of CD34, CD41 and CD45 on the cell surface enabled us to correlate immunophenotype, Mk clonogenic activity and hematopoietic gene expression. At both time points, we found that Mk colonies were confined to cells that expressed either or both CD41 and CD34, with the highest frequency observed in cells that co expressed both surface markers. There was evidence of ongoing maturation of Mk in the collagen based clonal assays, with polyploidization and shedding of platelet-like particles. The platelet-like particles generated in these cultures displayed characteristics similar to platelets derived from cord blood platelets, including responsiveness to ADP.

CD41 (GpIIb) is expressed on a range of hematopoietic precursors during early hematopoietic development in the embryo, from ES cells and in the adult [16]–[19]. However, in the cytokine milieu present in our studies, after 13 days of differentiation, most of the clonogenic cells in the CD41^+^ fractions formed Mks. These observations are reminiscent of the findings in adult mouse and human bone marrow and cytokine-mobilized peripheral blood stem cells, in which Mk precursors were most enriched in CD41^+^CD34^+^ cells [19], [29]–[31]. However, others have suggested that it was CD41^−^CD34^+^ cells from both cytokine-mobilized peripheral blood and umbilical cord blood that contained the most immature Mk progenitors with the greatest proliferative potential [32]. Follow up studies by the same authors examined the engraftment potential of TPO-expanded cord blood cells and demonstrated that the most rapid emergence of human platelets in the peripheral blood of immunocompromised mice followed the transplantation of CD34^−^CD61^−^ cells, rather than the transplantation of cells expressing identifiable Mk surface markers [33]. It is interesting to note that the phenotypic development of Mk from cytokine-mobilized peripheral blood, in which Mk transiently co-expressed CD34 and CD41, differed from Mk development from umbilical cord blood, in which Mk progenitors transiently lost CD34 expression before acquiring CD41 [32]. In our experiments we also observed a tendency for larger Mk colonies to be formed by d13 CD41^−^CD34^+^ cells and d20 CD41^lo/−^CD34^+^CD45^−^ cells (Figure 3C, 3F). The fact that we observed Mk progenitors in CD41^+^CD34^+^ populations and no clonogenic activity in CD41^−^CD34^−^ cells suggests that our differentiating hESC cultures were more similar in their behavior to cytokine-mobilized peripheral blood.

Generation of Mks and their precursors from hESCs has been demonstrated in several studies in which co-culture of differentiating hESC with OP9 mouse stromal cells was a common feature. Erythroid-megakaryocyte progenitors were first identified in a CD43^+^CD41a/CD235^+^ subset of cells after 6 days of differentiation [20]. In a later study from another laboratory, a similar erythroid-megakaryocytic precursor present from day 8 of hESC differentiation that co-expressed CD235, CD41a, CD43 and CD34, could be cultured to generate CD235^−^CD41a^+^ Mk progenitors and CD235^+^CD41a^−^ erythroid progenitors [7]. Megakaryocytes co-expressing CD41a and CD42 were observed after a 2 week differentiation on OP9 stromal cells in serum containing medium supplemented with TPO, although the phenotype of the progenitor population was not determined in this study [34]. In these cultures, even though a high proportion of Mk were polyploid, proplatelet formation was infrequently observed [34].

Culturing hESC on C3H10T1/2 or OP9 feeder layers in serum containing medium supplemented with exogenous VEGF, Takayama and colleagues observed sac-like structures after two weeks that contained hematopoietic cells, approximately 13% of which co-expressed CD34 and CD41a [6]. Further co-culture of the isolated hematopoietic cells on fresh feeder cells for 9 days led to ∼50% cells co-expressing CD41a and CD42b and eventually the generation of platelet like particles in the culture supernatant. Double positive CD41a^+^CD42b^+^ cells could be generated from all combinations of CD41a and CD34 expressing sac cells, although fewer arose from the CD34^+^CD41a^−^ subpopulation [6]. Our observations that Mk colonies were confined to cells that expressed either or both CD41a and CD34 are consistent with these data.

It has been recognized that platelets derived from hiPSCs could be a useful source of patient matched platelets, circumventing the loss of responsiveness to platelet transfusions associated with immunorejection of non-autologous platelets. To explore this possibility, Takayama and colleagues generated Mk from hiPSCs via hematopoietic sacs, using the co-culture protocol they reported previously [35]. They serendipitously observed that reactivation of c*MYC*, one of the reprogramming genes introduced during iPSC generation, was associated with enhanced generation of Mk progenitors but that persistent c*MYC* expression reduced Mk differentiation efficiency [35]. They complemented these observations by demonstrating that enhanced platelet production was achieved by transient elevation, followed by reduction, of c*MYC* expression. Functional studies of iPSC-generated platelets showed that they responded to ADP activation by binding to PAC-1, spreading on fibrinogen and that transfused platelets adhered to thrombus in vivo. However, this form of platelet production might not be available to most patients needing platelets.

Lu and colleagues cultured differentiated hESCs in methylcellulose to form hematopoietic blast colonies and directed their further differentiation towards the Mk lineage by culturing in medium supplemented with TPO, SCF and IL-11 [8]. However, the progenitor population that developed into CD41a^+^ CD42b^+^ Mks after a few days was not identified. Interestingly, only ∼5% of the hESC-derived platelets generated under these feeder- and serum-free conditions co-expressed CD41a and CD42b. Co-culture of Mk with OP9 stromal cells in medium supplemented with TPO, SCF and sodium heparin increased the proportion of CD41a^+^CD42b^+^ cells to ∼40%. These hESC-derived Mks produced platelets that were activated by thrombin, spread on fibrinogen- and vWF-coated surfaces and formed fibrin clots.

In conclusion, our study has demonstrated that CD34^+^ and CD41^+^ cells differentiated from hESCs in serum-free, feeder cell-free culture, generated clonogenic Mk progenitors in response to thrombopoietic combinations of cytokines. This work complements previous studies that demonstrated the feasibility of generating functional platelets from human pluripotent cells, and represents a further step towards the generation of human platelets under defined conditions for therapeutic use in the future.

## Supporting Information

Figure S1 **Staining patterns of CD41 on cultures of differentiating hESCs.** Dot plots showing SSC and CD41 expression on day 13 and 20 cultures of differentiating hESC compared to staining with isotype control antibodies.
(DOCX)

Figure S2 **Hematopoietic colonies generated in methylcellulose from day 13 and 20 differentiated hESCs sorted on CD34, CD41 and CD45.** Histograms show myeloid and erythroid colony frequency displayed as mean±SD, from n = 5 independent experiments.
(DOCX)

Table S1 **Distribution of CD34, CD41 and CD45 expressing cells sorted from differentiated hESCs HES 3 and cell lines.** Table represent the average distribution from five experiments of the percent CD34+ hematopoietic “stem” cells, CD41+ megakaryocytes and CD45+ total white blood cells generated in the differentiation of hESCs cells at both day 13 and day 20 of differentiation.
(DOCX)
