# Supplementary figures and images for: Generation of Megakaryocytic Progenitors from Human Embryonic Stem Cells in a Feeder- and Serum-Free Medium
Source: PLoS One. 2013 Feb 12;8(2):e55530. doi: 10.1371/journal.pone.0055530 (PMC3570533; doi:10.1371/journal.pone.0055530)

**Supplementary Figures & Figure legends:**


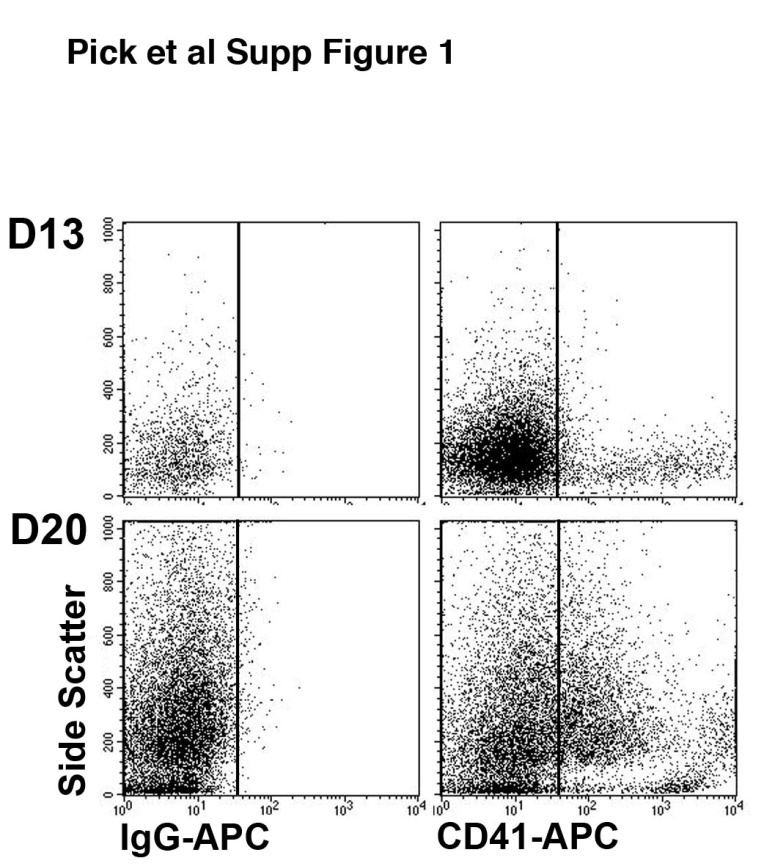

Supplement: Figure S1 — Staining patterns of CD41 on cultures of differentiating hESCs. Dot plots showing SSC and CD41 expression on day 13 and 20 cultures of differentiating hESC compared to staining with isotype control antibodies. (DOCX) [file pone.0055530.s001.docx]

**
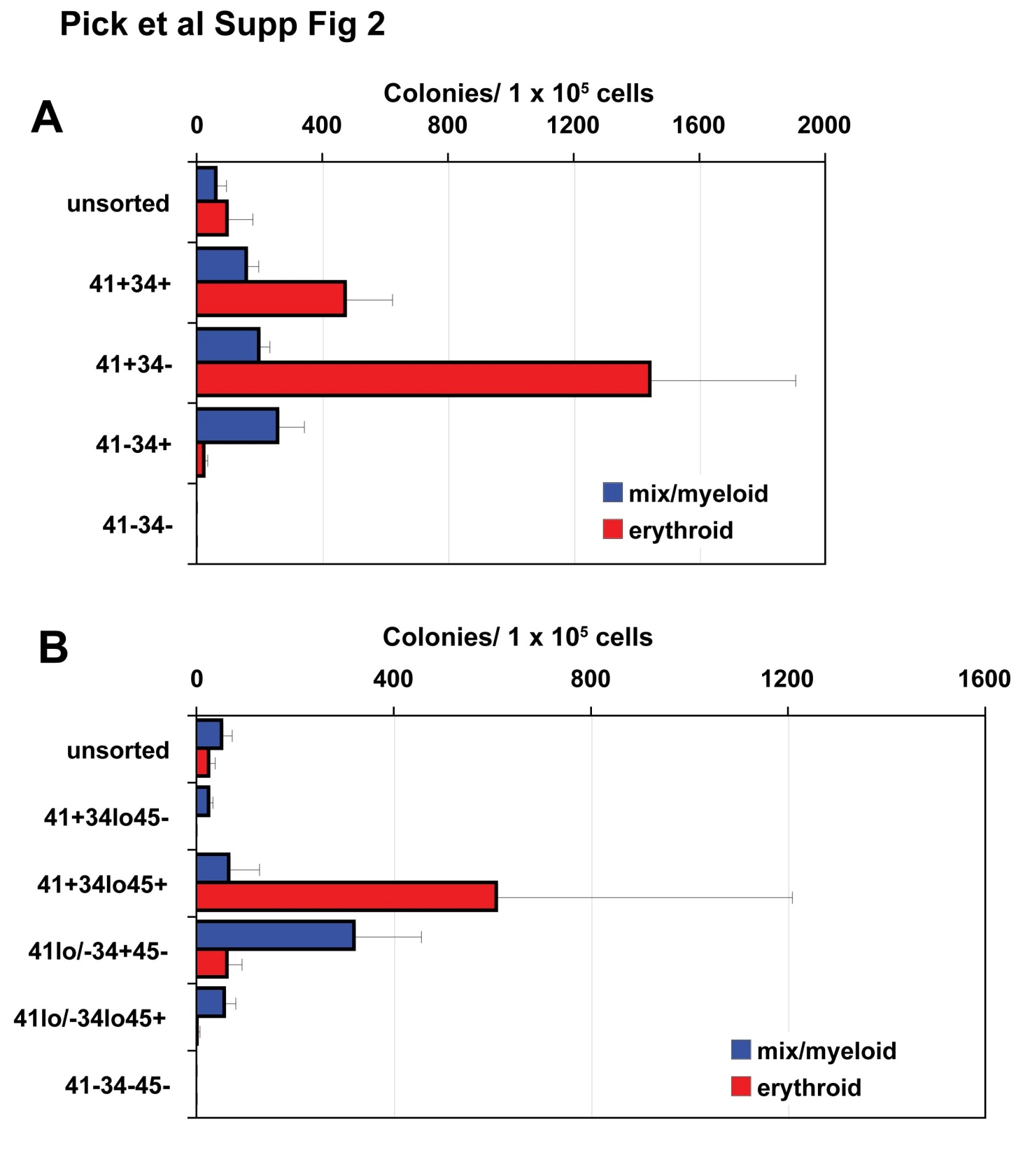
**

Supplement: Figure S2 — Hematopoietic colonies generated in methylcellulose from day 13 and 20 differentiated hESCs sorted on CD34, CD41 and CD45. Histograms show myeloid and erythroid colony frequency displayed as mean±SD, from n = 5 independent experiments. (DOCX) [file pone.0055530.s002.docx]
