# Supplementary material for: Generation of Megakaryocytic Progenitors from Human Embryonic Stem Cells in a Feeder- and Serum-Free Medium
Source: PLoS One. 2013 Feb 12;8(2):e55530. doi: 10.1371/journal.pone.0055530 (PMC3570533; doi:10.1371/journal.pone.0055530)
Supplement: Table S1 — Distribution of CD34, CD41 and CD45 expressing cells sorted from differentiated hESCs HES 3 and cell lines. Table represent the average distribution from five experiments of the percent CD34+ hematopoietic “stem” cells, CD41+ megakaryocytes and CD45+ total white blood cells generated in the differentiation of hESCs cells at both day 13 and day 20 of differentiation. (DOCX) [file pone.0055530.s003.docx]

**Table S1. Distribution of CD34, CD41 and CD45 expressing cells sorted from differentiated hESCs**

|  | CD41+CD34+ | **CD41+CD34-** | **CD41-CD34+** | **CD41-CD34-** |  |
| --- | --- | --- | --- | --- | --- |
| D13 |  |  |  |  |  |
| HES3 | 1.4+0.68 | 1.3+0.77 | 5.3+3.33 | 91.9+3.54 |  |
| Envy | 1.5+0.90 | 1.2+0.53 | 6.7+3.94 | 90.6+4.93 |  |
|  |  |  |  |  |  |
|  | **CD41+CD34lo**  **CD45+** | **CD41lo/-CD34+**  **CD45-** | **CD41lo/-CD45+**  **CD34+/-** | **CD41+CD34lo**  **CD45-** | **CD41-CD34-**  **CD45-** |
| D20 |  |  |  |  |  |
| HES3 | 1.2+0.40 | 1.4+0.20 | 39.8+0.87 | 1.4+0.32 | 59.7+8.69 |
| Envy | 2.8+0.78 | 3.2+2.46 | 22.5+8.11 | 1.6+0.83 | 69.8+5.08 |

Values represent the percent mean+SD for each fraction evaluated before sorting was performed, n=5
